# An eye on equity: faricimab-driven health equity improvements in diabetic macular oedema using a distributional cost-effectiveness analysis from a UK societal perspective

**DOI:** 10.1038/s41433-024-03043-y

**Published:** 2024-03-30

**Authors:** Aurelie Meunier, Oyin Opeifa, Louise Longworth, Oliver Cox, Christian Bührer, Isabelle Durand-Zaleski, Simon P. Kelly, Richard P. Gale

**Affiliations:** 1 Putnam, London, UK; 2 F. Hoffmann-La Roche Ltd, Grenzacherstrasse, Basel, Switzerland; 3 AP-HP URCEco Hôtel Dieu, Université Paris Est Créteil, Paris, France; 4 Beaumont Hospital, Bolton, UK; 5 Hull York Medical School, York and Scarborough Teaching Hospitals NHS Foundation Trust, York, UK

**Keywords:** Eye diseases, Health care economics, Oedema

## Abstract

**Background/Objectives:**

Diabetic macular oedema (DMO) is a leading cause of blindness in developed countries, with significant disease burden associated with socio-economic deprivation. Distributional cost-effectiveness analysis (DCEA) allows evaluation of health equity impacts of interventions, estimation of how health outcomes and costs are distributed in the population, and assessments of potential trade-offs between health maximisation and equity. We conducted an aggregate DCEA to determine the equity impact of faricimab.

**Methods:**

Data on health outcomes and costs were derived from a cost-effectiveness model of faricimab compared with ranibizumab, aflibercept and off-label bevacizumab using a societal perspective in the base case and a healthcare payer perspective in scenario analysis. Health gains and health opportunity costs were distributed across socio-economic subgroups. Health and equity impacts, measured using the Atkinson inequality index, were assessed visually on an equity-efficiency impact plane and combined into a measure of societal welfare.

**Results:**

At an opportunity cost threshold of £20,000/quality-adjusted life year (QALY), faricimab displayed an increase in net health benefits against all comparators and was found to improve equity. The equity impact increased the greater the concerns for reducing health inequalities over maximising population health. Using a healthcare payer perspective, faricimab was equity improving in most scenarios.

**Conclusions:**

Long-acting therapies with fewer injections, such as faricimab, may reduce costs, improve health outcomes and increase health equity. Extended economic evaluation frameworks capturing additional value elements, such as DCEA, enable a more comprehensive valuation of interventions, which is of relevance to decision-makers, healthcare professionals and patients.

## Introduction

Diabetic macular oedema (DMO) is a complication of diabetic retinopathy (DR) affecting around 7% of diabetic patients in England [1]. DMO is one of the leading causes of blindness among people of working age in developed countries [2], and prevalence trends for diabetes and diabetic eye conditions are on the rise [3]. Based on the National Diabetes Audit, the prevalence of diabetes—and thus DMO—is higher in more socio-economically deprived areas of England, driven by type 2 diabetes [4].

In many jurisdictions, decision-making about funding new healthcare interventions is informed by a cost-effectiveness analysis (CEA) that seeks to maximise population health under budget constraints. Additional elements of value, which are often overlooked or underappreciated, have been proposed to broaden the valuation of pharmaceuticals; these include disease severity, societal costs (productivity, indirect costs), impact on caregivers, health equity or value of hope [5, 6]. CEAs can be augmented to incorporate a quantitative assessment of these additional value elements.

Health inequities are systematic, avoidable and unfair differences in health outcomes observed across populations [7]. Distributional cost-effectiveness analysis (DCEA) builds on CEA to quantify the health equity impact of recommending new interventions and evaluate potential trade-offs between health maximisation and reduction of unfair variations in health.

The National Institute for Health and Care Excellence (NICE) have set out to tackle disparities in health in England, making it a priority in their strategy for the period of 2021–2026 [8]. Implementation of DCEA is currently being explored by NICE to support the development of technology appraisal (TA) guidance.

We aimed to conduct an aggregate DCEA from an English National Health Service (NHS) perspective to determine the equity impact of faricimab for the treatment of DMO and capture its value more comprehensively.

## Diabetic macular oedema burden and equity

### Diabetes

An estimated 3.2 million people are living with diabetes in England, based on the latest National Diabetes Audit [4, 9]. Diabetes is more prevalent in areas of higher deprivation due to risk factors such as obesity, physical inactivity, smoking and low birth weight all associated with low socio-economic status [10]. Based on the National Diabetes Audit, 24% of people from the most deprived quintiles have type 2 diabetes, compared with 15% for people from the least-deprived areas [4]. There was no difference in type 1 diabetes prevalence by socio-economic deprivation [4]. Diabetes, particularly type 2 diabetes, disproportionately affects ethnic minorities within the United Kingdom (UK), including Asian and Black ethnic groups [11–13]. People from Asian (15.7%) and Black (15.2%) ethnic groups are more likely to be living in England’s most deprived 10% of neighbourhoods compared with their White counterparts (9.0%) [14], suggesting that deprivation is associated with diabetes.

Diabetes management requires frequent appointments with healthcare professionals, which may be challenging for patients of working age. Additionally, deprivation is documented to negatively impact access to health services. The 2020–21 National Diabetes Audit found that deprivation was associated with a reduced likelihood of receiving all eight care processes for diabetes prevention and management [15]. Due to poorer disease management, patients from more deprived neighbourhoods are more likely to have poor glycaemic control [16], increasing risks of diabetic complications such as diabetic kidney disease, cardiovascular disorders and diabetic eye disease [17, 18].

Along with physical comorbidities, patients with diabetes often have mental health challenges. A systematic review showed patients with type 2 diabetes had a 25% greater risk of depression than the general population, and 40% of patients with diabetes also had anxiety symptoms [19].

### Diabetic retinopathy and diabetic macular oedema

DR and DMO are ocular complications of diabetes leading to vision impairment. Simply stated, DMO is caused by swelling of the macula. Diabetic eye disease is one of the leading causes of blindness in adults of working age in most developed economies [2, 20], and the second most likely cause of blindness after inherited retinal disorders in England and Wales [21].

DMO is more prevalent among patients living in areas of higher deprivation because of the link with diabetes and risk factors such as poor glycaemic control and longer duration of disease, which may be more prevalent in lower socio-economic groups [16, 22].

A recent large real-world study in the UK showed that patients with diabetes living in more deprived areas are more likely to present with severe sight impairment upon the first visit to hospital eye services compared with those in more affluent areas [23].

The Diabetic Eye Screening Programme of the NHS provides regular photographic eye checks to diabetes patients over the age of 12, with the aim of early detection and referral for signs of DR, damages to the retina, to prevent progression to a sight-threatening stage [24]. One to two million patients with diabetes are screened in England each year [25, 26], avoiding vision loss and blindness for many [27]. However, there are still high levels of non-attendance, with uptake varying between 70% and 80% [25, 26], particularly in areas of higher deprivation as demonstrated in multiple retrospective real-world studies [28–30].

### Diabetic macular oedema management and treatment

Optimal management of diabetic eye disease can be achieved by early diagnosis via screening programmes and holistic care of diabetes. A multidisciplinary team of healthcare professionals including diabetologists, ophthalmologists, general practitioners and diabetes specialist nurses is needed to provide coordinated care, supporting and educating patients to optimise glycaemic and blood pressure control to reduce the risk of diabetes complications [31].

Anti-vascular endothelial growth factor (anti-VEGF) administered by intravitreal injection is the most common first-line therapy in DMO for patients with central retinal thickness ≥400 μm [32]. The burden of intravitreal injection and requirement for life-long therapy due to the chronic nature of the disease, with frequent administrations at treatment initiation although this tends to reduce in the long-term, create a significant treatment burden to patients. This may result in a barrier to treatment adherence and persistence in clinical practice with consequences on real-world health outcomes; however, this is not typically reflected in randomised clinical trials. A real-world evidence study investigated the usage of aflibercept in UK clinical practice and found rates of loss to follow-up of 10% at year one and 29% at year three [33]. Additionally, they reported that socio-economic deprivation was associated with higher rates of loss to follow-up but not the number of injections received or improvement in visual acuity [33]. Another retrospective study investigating treatment patterns with anti-VEGF reported treatment discontinuation of 28% after 2 years [34]. Similar patterns have been observed across Europe and the United States [35–37].

Frequent injections create capacity constraints on ophthalmology services, which are the busiest outpatient speciality in NHS England [38], leading to delayed treatment or increased costs for healthcare services to manage such demand, including private clinics or out-of-hour additional clinical session costs. Since the 2008 introduction of ranibizumab, the first anti-VEGF therapy recommended by NICE, the number of injections to treat DMO and other eye disorders in NHS England is on the rise, with an estimated increase of 215% between 2010–2011 and 2014–2015. The cost of ranibizumab and aflibercept was estimated to be £447 million in 2015–2016, not accounting for commercial in confidence discount rates [39].

Faricimab (Vabysmo®) is the first dual-pathway-inhibitor of angiopoietin-2 and vascular endothelial growth factor-A designed for intraocular use. It demonstrated sustained efficacy using a treat and extend (T&E) regimen with an initial loading phase followed by individualised dosing intervals of up to 16 weeks compared with aflibercept (Eylea®) administered every 8 weeks in two non-inferiority phase 3 double-blind randomised trials (YOSEMITE and RHINE) [40]. NICE recommended faricimab for the treatment of DMO for patients with central retinal thickness ≥400 μm in 2022 and listed it as an opportunity to increase productivity of the healthcare system, via a reduction in clinical appointments [41].

Faricimab T&E dosing may reduce eye treatment burden for patients and healthcare systems, with the potential to reduce costs, improve health outcomes in clinical practice and improve health equity.

## Distributional cost-effectiveness analysis methods

Funding new interventions in a healthcare setting managed under a fixed budget is achieved by disinvesting other services. Changes in population health are thus the result of the health benefits derived from the new funded therapies and the health losses due to the disinvestment of services, also called health opportunity cost. CEA is concerned with efficiency, looking at the average impact of introducing new interventions in the system. In a DCEA, the underlying objective is to evaluate which population groups “gains” and which “losses” and by how much. This is achieved by first estimating the distribution of the health benefits and health opportunity costs, which are summed up to get the distribution of the net health benefit (NHB). This is the distributional impact of the new intervention, which is added to a baseline health distribution to assess the resulting changes in the distribution of population health. Then, equity impacts and potential trade-offs between health maximisation and reduction of health inequalities can be evaluated [42]. A schematic diagram of the method is presented in in the Supplementary Materials (A2).

An aggregate DCEA approach was adopted using average costs and health outcomes from a CEA to derive the distributions of the health benefits and health opportunity costs in the population [43].

### Simulation of population health distributions

Differences in health of concern in the present analysis were those between socio-economic subgroups. The English index of multiple deprivation (IMD) measures relative levels of socio-economic deprivation for small areas in England (about 1500 residents), combining seven domains of deprivation (income, employment, education, health, crime, housing and living environment). The population is grouped into five quintiles.

The baseline distribution of population health by IMD quintiles was taken from a study by Love-Koh et al. [44], who estimated the social distribution of quality-adjusted life expectancy (QALE) at birth in England.

Data on health benefits and costs were derived from a CEA conducted by F. Hoffman-La Roche Ltd, which is described in full elsewhere [45]. The CEA uses an English NHS and personal social services perspective to compare faricimab (28.8 mg vial = £857.00) [46] with ranibizumab (2.3 mg vial = £551.00) [47] and aflibercept (4.0 mg vial = £816.00) [48], which are treatments recommended by NICE [49–51], and off-label bevacizumab (100.0 mg vial = £242.66) [52], based on cost per vial (no vial sharing based on the drug labels in England) (Table 1). In line with UK clinical practice, aflibercept and ranibizumab are administered every 4 weeks during an initial loading phase and then as required (pro re nata [PRN]). An additional T&E ranibizumab comparator was included in scenario analysis because this regimen is sometimes used in NHS England. Despite intravitreal bevacizumab not being licensed for the treatment of any retinal condition, including DMO, it is commonly used off label in international clinical practice because of its low price. Off-label bevacizumab PRN is occasionally used to treat patients with DMO in NHS hospitals in England and was included as a scenario analysis. Effectiveness data were informed by the YOSEMITE and RHINE randomised trials and a network meta-analysis [40, 51]. It was estimated that 8.4–9.9 injections are required in the first year depending on the regimen, 4.9–5.5 in the second year and around 2 injections from year three onwards. Details are presented in the Supplementary Materials (A1). A societal perspective, including productivity gains and informal care costs, was adopted in the base case and a healthcare payer perspective was explored in a sensitivity analysis.

**Table 1:** Health benefits and costs inputs based on a cost-effectiveness model of faricimab.

	Faricimab T&E vs ranibizumab PRN	Faricimab T&E vs aflibercept PRN	Faricimab T&E vs off-label bevacizumab PRN	Faricimab T&E vs ranibizumab T&E
Incremental QALYs	0.36	0.16	0.39	0.36
Societal perspective				
Incremental costs	−£5483	−£9655	£3478	−£5478
Incremental net health benefit	0.63	0.64	0.22	0.63
Healthcare payer perspective				
Incremental costs	£2854	−£5498	£6518	−£2438
Incremental net health benefit	0.22	0.43	0.06	0.48

The cost-effectiveness model underlying these inputs was developed by F. Hoffman-La Roche Ltd (Basel, Switzerland). Incremental net health benefits have been calculated based on the base-case opportunity cost threshold of £20,000 per quality-adjusted life year.

*QALY* quality-adjusted life year, *PRN* pro re nata, *T&E* treat and extend.

Data on the target patient population were extracted from a combination of sources (Table 2). Diabetes prevalence data in the adult population were informed by the Quality and Outcomes Framework 2021–2022 [9], and diabetes cases by IMD were taken from the National Diabetes Audit [4]. DMO prevalence statistics were informed by literature [1]. The eligibility criteria for faricimab were taken from the NICE TA [51].

**Table 2:** Population/disease distributional inputs.

	IMD1 (most deprived)	IMD2	IMD3	IMD4	IMD5 (least deprived)	Source
Baseline distribution of health (QALE)	63.21	67.61	69.95	73.10	75.00	Love-Koh et al. [44]
Health opportunity costs distribution	26%	22%	22%	16%	14%	Love-Koh et al. [54]
Proportion of DMO cases	24%	22%	21%	18%	15%	Calculated (see Supplementary Materials A2)

*DMO* diabetic macular oedema, *IMD* index of multiple deprivation, *QALE* quality-adjusted life expectancy.

The opportunity cost threshold, representing the cost per quality-adjusted life year (QALY) foregone because of displacing resources in the NHS, was aligned with the lower bound of the standard NICE cost-effectiveness threshold range: £20,000/QALY, in line with previous studies [42]. Sensitivity analyses were conducted, including a threshold value of £15,000/QALY, used by the English Department for Health and Social Care [53] and £30,000/QALY, the upper bound of the NICE threshold range. The distribution of health opportunity costs by IMD was informed by literature [54].

### Evaluation of equity impacts and potential trade-offs

An equity-efficiency impact plane was plotted, where the *x*-axis was the population health equity impact and the *y*-axis was the incremental population NHB, to map out potential trade-offs between health and equity maximisation. Equity impacts were measured using the Atkinson inequality index [55], which explicitly captures societal preferences to forego some of the population health to reduce health inequalities, through an inequality aversion parameter (IAP). Based on a recent study, the Atkinson IAP for the general population of England is of 10.95 (95% confidence interval, 10.95–10.95), implying that health benefits accrued in the most deprived quintiles were valued seven times as highly as health benefits in the least-deprived quintiles. The equally distributed equivalent health (EDEH) of a distribution is the level of health per person that, if equally distributed across the population, would give the same level of societal welfare as the current unequal distribution [56]. An illustration of the EDEH is presented in the Supplementary Materials (A2). EDEH is a measure of societal welfare calculated by combining the Atkinson index with the mean level of health in the distribution, providing an equity-weighted summary measure of the population distribution of health.

Scenario analyses were conducted altering the health opportunity costs threshold, the IAP and the cost-effectiveness model perspective.

## Results

It was estimated that visual impairment due to DMO in England affects 86,393 people, of whom 4179 would be eligible for treatment with faricimab T&E each year; 24% would live in the most deprived areas (IMD1) and 15% in the most affluent (IMD5). Detailed calculations are presented in the Supplementary Materials (A3).

**Fig. 1:**
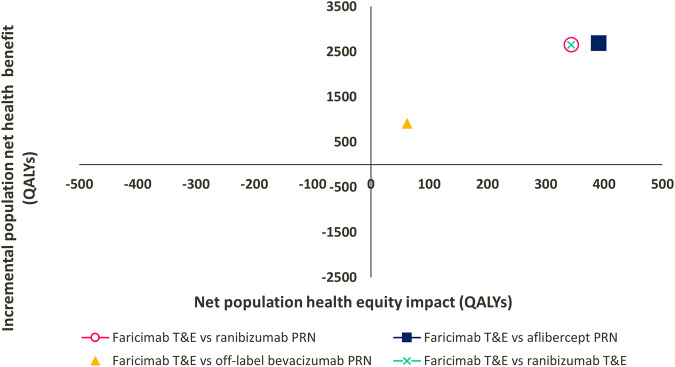
Equity-efficiency impact plane of faricimab against comparator treatments at base case. PRN pro re nata; QALY quality-adjusted life year; T&E treat and extend. Analysis settings: societal perspective, £20,000/QALY opportunity cost threshold; Atkinson inequality aversion parameter = 10.95.

Plotted on the equity-efficiency impact plane faricimab T&E was both health and equity improving against all comparators under the base-case assumptions (top-right quadrant) (Fig. 1).

Compared with ranibizumab PRN, faricimab T&E displayed an increase in population NHB, of 2650 QALYs, an increase in societal welfare (capturing changes in both health and equity), measured by the change in EDEH, of 2994 QALYs, and an equity impact (difference between the equity-weighted population health [EDEH] and the population health [QALE]) of 344 QALYs, representing the increase in societal welfare attributable to the reduction of inequalities (Table 3). Compared with aflibercept PRN, the reduction of health inequalities was equivalent to 391 QALYs.

**Table 3:** Base-case population health, societal welfare and equity impacts.

	Base case		Scenarios	
	Faricimab T&E vs ranibizumab PRN	Faricimab T&E vs aflibercept PRN	Faricimab T&E vs off-label bevacizumab PRN	Faricimab T&E vs ranibizumab T&E
Evaluating changes in population health (change in equity not included)				
Baseline population QALE (QALE_b_*N) (1)	3,939,130,276 QALYs	3,939,130,276 QALYs	3,939,130,276 QALYs	3,939,130,276 QALYs
Post-decision population QALE (QALE_p_*N) (2)	3,939,132,926 QALYs	3,939,132,962 QALYs	3,939,131,179 QALYs	3,939,132,925 QALYs
Incremental population QALE (=incremental NHB) (∆QALE*N) (3) = (2) − (1)	2650 QALYs	2686 QALYs	903 QALYs	2649 QALYs
Evaluating changes in equity-weighted health (changes in health and health equity both included)				
Baseline population EDEH (equity weighted QALE) (EDEH_b_*N) (4)	3,860,269,446 QALYs	3,860,269,446 QALYs	3,860,269,446 QALYs	3,860,269,446 QALYs
Post-decision population EDEH (EDEH_p_*N) (5)	3,860,272,440 QALYs	3,860,272,523 QALYs	3,860,270,411 QALYs	3,860,272,439 QALYs
Incremental population EDEH (∆EDEH*N) (6)	2994 QALYs	3077 QALYs	965 QALYs	2993 QALYs
Health equity impact				
Population equity impact (incremental EDEH − incremental QALE) (6 − 3)	344 QALYs	391 QALYs	62 QALYs	344 QALYs

Analysis settings: societal perspective, £20,000/QALY opportunity cost threshold, Atkinson inequality aversion parameter = 10.95.

*QALE*_*b*_ baseline quality-adjusted life expectancy at birth per person, *QALE*_*p*_ post-decision quality-adjusted life expectancy at birth per person, *∆QALE* difference in QALE between post-decision and baseline, *EDEH*_*b*_ baseline equally distributed equivalent health per person, *EDEH*_*p*_ post-decision equally distributed equivalent health per person, *∆EDEH* difference in EDEH between post-decision and baseline, *N* England population, *PRN* pro re nata, *T&E* treat and extend, *QALY* quality-adjusted life year.

Scenario analyses demonstrated that faricimab T&E was both health and equity improving compared with off-label bevacizumab PRN and ranibizumab T&E, leading to an increase in societal welfare and a reduction of health inequalities of 62 QALYs and 344 QALYs, respectively.

**Fig. 2:**
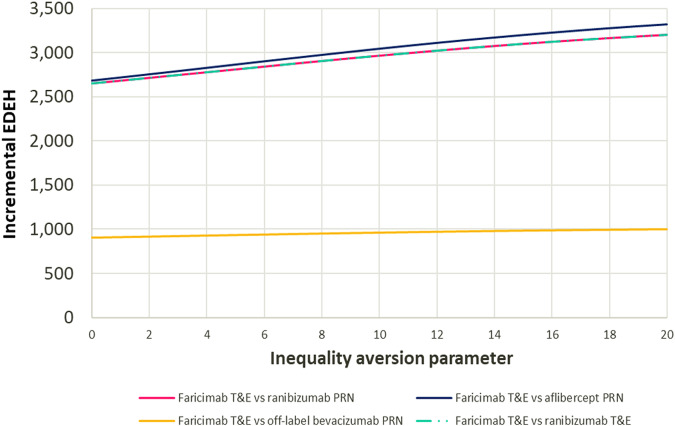
Equity-weighted population health impact with increasing inequality aversion parameter at base case. EDEH equally distributed equivalent health; PRN pro re nata; QALE quality-adjusted life expectancy; T&E treat and extend. Analysis settings: societal perspective, £20,000/QALY opportunity cost threshold. Greater value of the inequality aversion parameter reflects a higher willingness to trade some of the population health to reduce health inequalities.

Varying the Atkinson IAP between 0 (no equity weighting, incremental EDEH = incremental NHB) and 20, the equity impact of faricimab was positive and increased with larger value of IAP, as health gains among the most deprived are valued more highly (Fig. 2).

Varying the opportunity cost threshold changed the size of the health and equity impacts but not the direction, faricimab T&E remained health and equity improving against all four comparators. Detailed results are presented in Supplementary Materials (A5). Under a healthcare perspective and at an opportunity cost threshold of £20,000/QALY, faricimab T&E was health and equity improving against ranibizumab PRN, aflibercept PRN and ranibizumab T&E. Compared with bevacizumab PRN, which is infrequently used off label in NHS care, faricimab T&E involved a trade-off between health maximisation and reduction of health inequalities. At the base-case Atkinson IAP value of 10.95, faricimab improved societal welfare against all four comparators. Detailed results of adopting a healthcare payer perspective are presented in Supplementary Materials (A6).

## Discussion

We assessed the equity impact of adopting faricimab in NHS England compared to existing treatments. We found that faricimab T&E was health and equity improving against all comparators under a societal perspective and at an opportunity cost threshold of £20,000/QALY, driven by faricimab T&E having a positive incremental NHB and the patient distribution being skewed toward the most deprived quintiles. The results were sensitive to the value of the opportunity cost threshold despite the small patient numbers. In a scenario analysis, using a healthcare payer perspective, faricimab T&E compared with off-label bevacizumab PRN improved societal welfare, implying that the increase in total population health compensated for the increase in health inequities at the chosen level of equity aversion.

We attempted to replicate the standard of care in NHS clinical practice in England, including a range of treatments and dosing regimens, of which bevacizumab PRN which is available at low cost and used off label in ophthalmology departments occasionally. The illustrative example of an aggregate DCEA presented here aimed to capture the value of faricimab T&E for the treatment of DMO in England more comprehensively, demonstrating how equity impacts can be quantified to provide additional information to decision-makers, patients and healthcare providers.

This study has some limitations. First, patient access schemes for the intervention and comparators were not included in the underlying cost-effectiveness model, given that they are commercial in confidence. Including discounts would have reduced the acquisition costs of faricimab and the comparators which could lead to larger or smaller incremental costs and NHBs, with an unknown effect on the direction and size of the equity impact. Second, we conducted a DCEA in its aggregate form, using average health benefits and costs (assumed equal across all IMD subgroups) and incorporating IMD-specific parameters only in the estimation of the target population. The analysis gave a ballpark estimate of the size and direction of the equity impact of faricimab T&E to initiate discussions on equity concerns. We encourage researchers to perform similar analyses for emerging therapies or regimens for the treatment of DMO and new treatments generally.

Studies have demonstrated that treatment uptake is an important mediating factor to improve population health and reduce health inequities [57, 58]. Patients living with multiple physical and mental health comorbidities and social challenges are more likely to be unable to attend for frequent injections; patients who are adherent to DMO treatments experience larger gains in visual acuity [59]. Therefore, longer acting therapies with less burdensome dosing schedules, such as faricimab T&E, have the potential to improve treatment uptake, thereby improving outcomes and equity. Treatment discontinuation was assumed to be equal across treatments in the underlying CEA used as the basis of this DCEA, which could be a conservative scenario, underestimating the potential benefit of faricimab. As detailed in Section “Diabetic macular oedema burden and equity”, socio-economic deprivation is associated with DMO on the continuum of the disease and treatment pathway, including disease prevalence, severity and treatment burden. Further research applying a full DCEA methodology, whereby cost-effectiveness model parameters (e.g., visual equity at baseline, treatment adherence) are customised for each subgroup to derive IMD-subgroup specific health outcomes and cost estimates, could be done to home in on equity issues discussed above and refine the estimates of equity impacts. Failure to incorporate gradients in model inputs may results in uncertainty in the estimates [58]; therefore, resources should be invested to collect and analyse data disaggregated by equity relevant characteristics, such as socio-economic deprivation, age, sex and ethnicity [60].

Ophthalmology is the busiest outpatient speciality in NHS England, and there is a shortage of ophthalmologists [38, 61], leading to many services working at full capacity. Ophthalmology services in England have been severely impacted by the COVID-19 pandemic, with a drop of 32% in outpatient attendance [38, 62], resulting in an estimated 600,000 patients awaiting treatment as of January 2023 [63]. Given the negative impact of delayed treatment on visual acuity [61, 64], with severe sight impairment having substantial impact on patients’ quality of life, healthcare costs and societal costs, there is an urgent need to clear the backlog of care. Innovative ophthalmology treatments that are effective and less resource intensive, such as faricimab T&E, would help mitigate capacity constraints. The number of injections avoided thanks to new treatments are routinely captured in economic evaluations. However, the number of injections that cannot be delivered because of capacity constraints, and associated impact on patients’ health and costs to the healthcare system, are often ignored. Augmenting economic evaluations to adopt a capacity-constraint perspective, such as that proposed by Gale et al. [65], would support a more comprehensive assessment of the value of durable treatments.

CEA is the mainstay of healthcare intervention value assessment, informing decision-making in many countries. However, it has been recognised to have limitations, and extended frameworks have been proposed to incorporate additional elements of value [5, 6]. Including a quantitative assessment of equity impacts using DCEA enables a more comprehensive assessment of value, which is relevant to health technology assessment bodies such as NICE that are committed to reducing health inequalities [8]. Methodologies to incorporate equity consideration into value assessment, such as DCEA, have gained greater attention in recent years, reflecting growing policy focus on health equity, accelerated by the COVID-19 pandemic. The continued development of extended CEA frameworks to capture additional elements of value of healthcare interventions such as equity, service capacity impact, disease severity and value of hope would be valuable to health technology assessment agencies, manufacturers, healthcare providers and patients.

Economic evaluations are powerful quantitative tools to rationalise decision-making. However, they are one of many drivers in healthcare prioritisation, which is a complex process influenced by a range of stakeholders, including patient groups, pharmaceutical companies, healthcare professionals and policy makers, each trying to maximise their interests and influence [66, 67]. In a healthcare system with limited resources, funding for new interventions is achieved by displacing resources. The present analysis aimed to estimate the distributional impacts (health gains and losses) of such decision on socio-economic subgroups. Claxton et al. [68] conducted a study evaluating the impact of changes to NHS budget in terms of foregone health by disease area, with the aim of estimating the cost per QALY at the margin in NHS England. They estimated that the budget impact of funding ranibizumab for the treatment of DMO in England would result in additional deaths and QALYs foregone mainly in circulatory, respiratory, gastrointestinal, cancer, neurological disorders and mental health [68]. Making tangible consequences of funding decisions and understanding the viewpoint and incentives of each stakeholder in the decision-making process, as well as the delivery of healthcare, are important to achieve multiple and sometimes conflicting healthcare prioritisation goals (e.g., health maximisation, reduction of unfair differences in health).

This study suggests that faricimab T&E is equity improving and cost-effective for the treatment of DMO. Patients living with multiple health and social challenges, as well as sight-threatening diabetic eye disease, are also those most unavailable to attend NHS treatments. The increased durability of intravitreal injections such as faricimab and T&E dosing, compared with conventional treatments, will create an opportunity to reduce the treatment burden for patients and healthcare systems, improving outcomes for the most vulnerable individuals in particular.

## Summary

### What was known before

- Diabetes and diabetic macular oedema (DMO), which is a leading cause of blindness among people of working age in developed countries, disproportionately affect those living in deprived areas of England. Socio-economic deprivation is associated with a significant burden in disease prevalence, disease severity, access and uptake of screening programmes and treatments for patients with diabetes and DMO. Anti-vascular endothelial growth factors are the most common first-line therapy for the treatment of DMO, requiring frequent intravitreal injections and imposing significant burden on patients and healthcare systems.

### What this study adds

- This study suggests that, under the base-case assumptions, faricimab treat and extend is both a cost-effective and equity-improving option for the treatment of DMO. Long-lasting therapies for the treatment of DMO, such as faricimab treat and extend, may give an opportunity to reduce the treatment burden and improve outcomes for patients, particularly for those facing multiple diabetes-related physical and mental health comorbidities often associated with socio-economic deprivation. Additional value elements (e.g., service capacity impact, disease severity, value of hope) can be captured by extended economic evaluation frameworks, such as DCEA does with equity, enabling a more comprehensive valuation of interventions, which is relevant to decision-makers, healthcare professionals and patients.

### Supplementary information

Supplementary materials

## Data Availability

Data sharing not applicable to this article as no datasets were generated or analysed during the current study.

## References

[1] Minassian DC, Owens DR, Reidy A (2012). Prevalence of diabetic macular oedema and related health and social care resource use in England. Br J Ophthalmol..

[2] Romero-Aroca P (2011). Managing diabetic macular edema: the leading cause of diabetes blindness. World J Diabetes..

[3] Haider S, Thayakaran R, Subramanian A, Toulis KA, Moore D, Price MJ (2021). Disease burden of diabetes, diabetic retinopathy and their future projections in the UK: cross-sectional analyses of a primary care database. BMJ Open..

[4] NHS Digital. National Diabetes Audit Programme. 2022. https://digital.nhs.uk/data-and-information/clinical-audits-and-registries/national-diabetes-audit.

[5] Lakdawalla DN, Doshi JA, Garrison LP, Phelps CE, Basu A, Danzon PM (2018). Defining elements of value in health care—a health economics approach: an ISPOR special task force report [3]. Value Health.

[6] Neumann PJ, Garrison LP, Willke RJ (2022). The history and future of the “ISPOR Value Flower”: addressing limitations of conventional cost-effectiveness analysis. Value Health..

[7] McCartney G, Popham F, McMaster R, Cumbers A (2019). Defining health and health inequalities. Public Health..

[8] NICE. NICE strategy 2021 to 2026. London: National Institute for Health and Care Excellence; 2021.

[9] NHS Digital. Data on file: quality and outcomes framework, 2021–22. 2022. https://digital.nhs.uk/data-and-information/publications/statistical/quality-and-outcomes-framework-achievement-prevalence-and-exceptions-data/2021-22.

[10] Connolly V, Unwin N, Sherriff P, Bilous R, Kelly W (2000). Diabetes prevalence and socioeconomic status: a population based study showing increased prevalence of type 2 diabetes mellitus in deprived areas. J Epidemiol Community Health..

[11] Hippisley-Cox J, Coupland C, Robson J, Sheikh A, Brindle P (2009). Predicting risk of type 2 diabetes in England and Wales: prospective derivation and validation of QDScore. BMJ..

[12] Mathur R, Farmer RE, Eastwood SV, Chaturvedi N, Douglas I, Smeeth L (2020). Ethnic disparities in initiation and intensification of diabetes treatment in adults with type 2 diabetes in the UK, 1990–2017: a cohort study. PLoS Med..

[13] Pham TM, Carpenter JR, Morris TP, Sharma M, Petersen I. Ethnic differences in the prevalence of type 2 diabetes diagnoses in the UK: cross-sectional analysis of the health improvement network primary care database. Clin Epidemiol. 2019;11:1081–8. 10.2147/CLEP.S227621 PMC6948201 32021464

[14] Gov.UK. Ethnicity facts and figures: people living in deprived neighbourhoods. 2020. https://www.ethnicity-facts-figures.service.gov.uk/uk-population-by-ethnicity/demographics/people-living-in-deprived-neighbourhoods/latest#full-page-history.

[15] NHS Digital. National Diabetes Audit 2021–22, report 1: care processes and treatment targets, detailed analysis report. 2023. https://digital.nhs.uk/data-and-information/publications/statistical/national-diabetes-audit/report-1-care-processes-and-treatment-targets-2021-22-full-report/health-ineq-1718-2122.

[16] Whyte MB, Hinton W, McGovern A, van Vlymen J, Ferreira F, Calderara S (2019). Disparities in glycaemic control, monitoring, and treatment of type 2 diabetes in England: a retrospective cohort analysis. PLoS Med.

[17] Boye KS, Thieu VT, Lage MJ, Miller H, Paczkowski R (2022). The association between sustained HbA1c control and long-term complications among individuals with type 2 diabetes: a retrospective study. Adv Ther..

[18] Wild SH, McKnight JA, McConnachie A, Lindsay RS (2010). Socioeconomic status and diabetes-related hospital admissions: a cross-sectional study of people with diagnosed diabetes. J Epidemiol Community Health..

[19] Tomic D, Shaw JE, Magliano DJ (2022). The burden and risks of emerging complications of diabetes mellitus. Nat Rev Endocrinol..

[20] Heath Jeffery RC, Mukhtar SA, McAllister IL, Morgan WH, Mackey DA, Chen FK (2021). Inherited retinal diseases are the most common cause of blindness in the working-age population in Australia. Ophthalmic Genet..

[21] Liew G, Michaelides M, Bunce C (2014). A comparison of the causes of blindness certifications in England and Wales in working age adults (16–64 years), 1999–2000 with 2009–2010. BMJ Open.

[22] Low L, Law JP, Hodson J, McAlpine R, O’Colmain U, MacEwen C (2015). Impact of socioeconomic deprivation on the development of diabetic retinopathy: a population-based, cross-sectional and longitudinal study over 12 years. BMJ Open..

[23] Denniston AK, Lee AY, Lee CS, Crabb DP, Bailey C, Lip PL (2019). United Kingdom Diabetic Retinopathy Electronic Medical Record (UK DR EMR) Users Group: report 4, real-world data on the impact of deprivation on the presentation of diabetic eye disease at hospital services. Br J Ophthalmol..

[24] Gov.UK. Diabetic eye screening: programme overview. 2014. https://www.gov.uk/guidance/diabetic-eye-screening-programme-overview.

[25] Gov.UK. NHS screening programmes in England: 2019 to 2020. 2023. https://www.gov.uk/government/publications/nhs-screening-programmes-annual-report/nhs-screening-programmes-in-england-2019-to-2020#nhs-diabetic-eye-screening-des-programme.

[26] Gov.UK. NHS screening programmes in England: 2020 to 2021. 2023. https://www.gov.uk/government/publications/nhs-screening-programmes-annual-report/nhs-screening-programmes-in-england-2020-to-2021.

[27] Scanlon PH (2021). The contribution of the English NHS Diabetic Eye Screening Programme to reductions in diabetes-related blindness, comparisons within Europe, and future challenges. Acta Diabetol..

[28] Waqar S, Bullen G, Chant S, Salman R, Vaidya B, Ling R (2012). Cost implications, deprivation and geodemographic segmentation analysis of non-attenders (DNA) in an established diabetic retinopathy screening programme. Diabet Metab Syndr..

[29] Thomas RL, Cheung WY, Rafferty JM, Luzio SD, Akbari A, Owens DR (2021). Characteristics of repeat non‐attenders at Diabetes Eye Screening Wales, a national community‐based diabetes‐related retinopathy screening service, during 2003–2018. Diabet Med.

[30] Olvera-Barrios A, Seltene M, Heeren TF, Chambers R, Bolter L, Tufail A (2021). Effect of ethnicity and other sociodemographic factors on attendance at diabetic eye screening: a 12-month retrospective cohort study. BMJ Open..

[31] Gale R, Scanlon PH, Evans M, Ghanchi F, Yang Y, Silvestri G (2017). Action on diabetic macular oedema: achieving optimal patient management in treating visual impairment due to diabetic eye disease. Eye..

[32] Virgili G, Parravano M, Evans J, Gordon I, Lucenteforte E (2017). Anti-vascular endothelial growth factor (anti-VEGF) drugs for diabetic macular oedema: a network meta‐analysis. Cochrane Database Syst Rev.

[33] Talks S, Stratton I, Peto T, Lotery A, Chakravarthy U, Eleftheriadis H (2022). Aflibercept in clinical practice; visual acuity, injection numbers and adherence to treatment, for diabetic macular oedema in 21 UK hospitals over 3 years. Eye..

[34] Peto T, Akerele T, Sagkriotis A, Zappacosta S, Clemens A, Chakravarthy U (2022). Treatment patterns and persistence rates with anti‐vascular endothelial growth factor treatment for diabetic macular oedema in the UK: a real‐world study. Diabet Med..

[35] Alvarez-Ramos P, Jimenez-Carmona S, Alemany-Marquez P, Cordoba-Doña JA, Aguilar-Diosdado M (2020). Socioeconomic deprivation and development of diabetic retinopathy in patients with type 1 diabetes mellitus. BMJ Open Diabetes Res Care.

[36] Nguyen CTN, Yosef M, Khalatbari S, Shah AR (2022). Sociodemographic variables associated with risk for diabetic retinopathy. Clin Diabetes Endocrinol..

[37] Patel D, Ananthakrishnan A, Lin T, Channa R, Liu TA, Wolf RM (2022). Social determinants of health and impact on screening, prevalence, and management of diabetic retinopathy in adults: a narrative review. J Clin Med..

[38] NHS Digital. Data on file. Hospital outpatient activity 2019–20: all attendances. 2020. https://digital.nhs.uk/data-and-information/publications/statistical/hospital-outpatient-activity/2019-20.

[39] Hollingworth W, Jones T, Reeves BC, Peto T (2017). A longitudinal study to assess the frequency and cost of antivascular endothelial therapy, and inequalities in access, in England between 2005 and 2015. BMJ Open.

[40] Wykoff CC, Abreu F, Adamis AP, Basu K, Eichenbaum DA, Haskova Z (2022). Efficacy, durability, and safety of intravitreal faricimab with extended dosing up to every 16 weeks in patients with diabetic macular oedema (YOSEMITE and RHINE): two randomised, double-masked, phase 3 trials. Lancet..

[41] NICE. Supporting the health and care system in improving productivity. 2023. https://www.nice.org.uk/productivity.

[42] Asaria M, Griffin S, Cookson R (2016). Distributional cost-effectiveness analysis: a tutorial. Med Decis Making.

[43] Love-Koh J, Cookson R, Gutacker N, Patton T, Griffin S (2019). Aggregate distributional cost-effectiveness analysis of health technologies. Value Health..

[44] Love-Koh J, Asaria M, Cookson R, Griffin S (2015). The social distribution of health: estimating quality-adjusted life expectancy in England. Value Health..

[45] Bührer C, Paling T, Gale R, Paulo T, Bagijn M. Cost-effectiveness of faricimab in the treatment of diabetic macular oedema (DMO): a UK analysis. Pharmacoecon Open. 2024. 10.1007/s41669-023-00465-4. 10.1007/s41669-023-00465-4 PMC11058163 38438829

[46] BNF. Medicinal forms: faricimab. 2022. https://bnf.nice.org.uk/drugs/faricimab-specialist-drug/medicinal-forms/.

[47] BNF. Ranimibuzab [specialist drug]. 2022. https://bnf.nice.org.uk/drugs/ranibizumab-specialist-drug/.

[48] BNF. Aflibercept [specialist drug]. 2022. https://bnf.nice.org.uk/drugs/aflibercept-specialist-drug/.

[49] NICE. Overview: ranibizumab for treating diabetic macular oedema. 2013. https://www.nice.org.uk/guidance/ta274.

[50] NICE. Overview: aflibercept solution for injection for treating wet age‑related macular degeneration. 2015. https://www.nice.org.uk/guidance/ta294.

[51] NICE. Faricimab for treating diabetic macular oedema (TA799). Technology appraisal guidance. 2022. www.nice.org.uk/guidance/ta799.

[52] BNF. Bevacizumab [specialist drug]. 2022. https://bnf.nice.org.uk/drugs/bevacizumab-specialist-drug/.

[53] Gov.UK. Autumn 2022 update to the Statutory Scheme controlling the costs of branded health service medicines. 2022. https://assets.publishing.service.gov.uk/government/uploads/system/uploads/attachment_data/file/1121627/2023-statutory-scheme-costs-of-branded-medicines-consultation-impact-assessment.pdf.

[54] Love-Koh J, Cookson R, Claxton K, Griffin S (2020). Estimating social variation in the health effects of changes in health care expenditure. Med Decis Making..

[55] Atkinson AB (1970). On the measurement of inequality. J Econ Theory.

[56] Robson M, Asaria M, Cookson R, Tsuchiya A, Ali S (2017). Eliciting the level of health inequality aversion in England. Health Econ..

[57] Paltiel AD, Schwartz JL, Zheng A, Walensky RP (2021). Clinical outcomes of a COVID-19 vaccine: implementation over efficacy. Health Aff..

[58] Yang F, Angus C, Duarte A, Gillespie D, Sculpher M, Walker S, et al. Comparing smoking cessation to screening and brief intervention for alcohol in distributional cost effectiveness analysis to explore the sensitivity of results to socioeconomic inequalities characterised in model inputs. York: University of York, Centre for Health Economics; 2021.

[59] Weiss M, Sim DA, Herold T, Schumann RG, Liegl R, Kern C (2018). Compliance and adherence of patients with diabetic macular edema to intravitreal anti-vascular endothelial growth factor therapy in daily practice. Retina..

[60] Meunier A, Longworth L, Kowal S, Ramagopalan S, Love-Koh J, Griffin S (2023). Distributional cost-effectiveness analysis of health technologies: data requirements and challenges. Value Health..

[61] The Royal College of Ophthalmologists. Census report: facing workforce shortages and backlogs in the aftermath of COVID-19: the 2022 census of the ophthalmology consultant, trainee and SAS workforce. London: The Royal College of Ophthalmologists; 2022.

[62] NHS Digital. Data on file. Hospital outpatient activity 2020–21: all attendances. 2020. https://digital.nhs.uk/data-and-information/publications/statistical/hospital-outpatient-activity/2020-21.

[63] NHSE. Data on file. Consultant-led referral to treatment waiting times data 2022–23. 2023. https://www.england.nhs.uk/statistics/statistical-work-areas/rtt-waiting-times/rtt-data-2022-23/.

[64] The Royal College of Ophthalmologists. Workforce census 2018. London: The Royal College of Ophthalmologists; 2018.

[65] Gale R, Cox O, Keenan C, Chakravarthy U (2022). Health technology assessment of new retinal treatments; the need to capture healthcare capacity issues. Eye..

[66] Durand-Zaleski I. Principles of cost-effectiveness studies and their use in haematology. Best Pract Res Clin Haematol. 2023;36:101441. 10.1016/j.beha.2023.101441 36907634

[67] Goddard M, Hauck K, Preker A, Smith PC (2006). Priority setting in health – a political economy perspective. Health Econ Policy Law..

[68] Claxton K, Martin S, Soares M, Rice N, Spackman E, Hinde S (2015). Methods for the estimation of the National Institute for Health and Care Excellence cost-effectiveness threshold. Health Technol Assess..

