# Supplementary material for: An eye on equity: faricimab-driven health equity improvements in diabetic macular oedema using a distributional cost-effectiveness analysis from a UK societal perspective
Source: Eye (Lond). 2024 Mar 30;38(10):1917–25. doi: 10.1038/s41433-024-03043-y (PMC11226444; doi:10.1038/s41433-024-03043-y)
Supplement: Supplementary file 1 — Supplementary materials [file 41433_2024_3043_MOESM1_ESM.docx]

**Manuscript for Eye Journal (Nature) – Supplementary materials**

## Title

An eye on equity: faricimab-driven health equity improvements in diabetic macular oedema using a distributional cost-effectiveness analysis from a UK societal perspective.

## Authors

Aurelie Meunier^1^, Oyin Opeifa^1^, Louise Longworth^1^, Oliver Cox^2^, Christian Bührer^2^, Isabelle Durand-Zaleski*^3^*, Simon P. Kelly^4^, Richard P. Gale^5^

1. Putnam, London, United Kingdom
2. F. Hoffmann-La Roche Ltd, Grenzacherstrasse, Basel, Switzerland
3. AP-HP URCEco Hôtel Dieu, Université Paris Est Créteil, Paris, France
4. Beaumont Hospital, Bolton, UK
5. Hull York Medical School, York and Scarborough Teaching Hospitals NHS Foundation Trust, York, UK

## Corresponding author

Aurelie Meunier

Putnam

22-24 Torrington Place

London

WC1E 7HJ

T: +44 20 3695 5802

E: aurelie.meunier@putassoc.com

ORCID number: 0000-0002-3018-9110

**A1. Cost-effectiveness model summary**

A health economic model was developed by Roche to compare the cost-effectiveness of faricimab treat and extended (T&E) against ranibizumab (when required [pro re nata]) PRN, aflibercept PRN, ranibizumab T&E and off-label bevacizumab PRN in patients with diabetic macular oedema (DMO). The analysis is summarised below and is reported in detail elsewhere [1].

The analysis was conducted from a UK healthcare perspective. Costs and outcomes were discounted at a rate of 3.5% per annum. The time horizon was 25 years, and the model outcomes were incremental costs per quality-adjusted life-year (QALY) gained. A societal perspective was adopted in the base-case, also including productivity gains and informal care costs, and a healthcare payer perspective (drug acquisition and administration, monitoring, adverse events, supportive care costs) was explored in a sensitivity analysis.

A cohort state-transition Markov model was developed consisting of 6 states relating to visual acuity combined with the treatment pathway states to measure the duration of treatment. Efficacy, safety and tolerability model inputs were based on the results of the YOSEMITE and RHINE trials, and a network meta-analysis for the comparators. Background mortality was informed by UK life tables from the Office of National Statistics. Treatment costs included drug acquisition and administration and were taken from the British National Formulary, UK clinical guidelines and the National Schedule of National Health Service (NHS) costs. Supportive care costs were sourced from a UK guideline and comprise low vision and blindness resources which fall upon NHS and Personal Social Services and wider societal costs (housing and council tax benefit, social security and governmental tax allowances). The costs of productivity losses/gains were estimated using a human capital approach. Utilities relating to visual acuity were based on literature while disutility from adverse effects was informed by a UK guideline.

The estimated number of injections for patients on treatment is presented in Figure S1.

Figure S1. Projected number of injections in the cost-effectiveness analysis

Note: Number of injections for patients on treatment. The analysis adjusts for discontinuation which is not reflected here. Estimates based on a cost-effectiveness analysis conducted by Roche-Ltd.

**A2. Distributional cost-effectiveness analysis methods**

**Methods overview**

Figure S2. Distributional cost-effectiveness analysis method overview


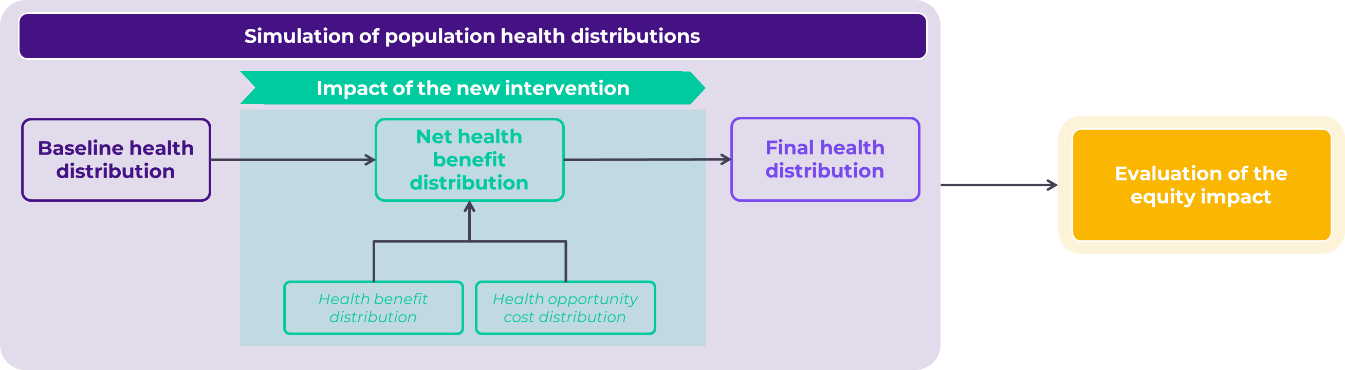


**Illustration of the equally distributed equivalent health**

Figure S3. Illustration of the equally distributed equivalent health

Abbreviations: IMD, index of multiple deprivation

A health distribution that is unequally distributed across the five IMD quintiles, with a mean value of 71 QALYs (no equity weighting) is presented in Figure S3., in purple. In yellow is the EDEH, the equity-weighted level of health which if equally distributed across all quintiles would provide the same level of societal welfare as the unequal distribution.

**A3. Distribution of eligible population**

Table S1 and Table S2 detail the calculations to estimate the distribution of DMO patients by IMD quintiles and the number of patients with DMO eligible for faricimab each year. An example is presented to show the number of patients living in the most deprived quintile (IMD1), eligible for treatment with faricimab.

Table S1. Distribution of diabetic macular oedema patients by index of multiple deprivation quintiles

|  | **Item** | **Patients** | **Source** |
| --- | --- | --- | --- |
| *Population estimates* | | | |
| A | England adult population | 44 715 461 | ONS [2] |
| *Diabetes prevalence data* | | | |
| B | Prevalence of diabetes in the adult population | 7.26% | NHS Digital [3] |
| C | % of which T1DM | 6.92% | NHS Digital [4] |
| D | % of which T2DM | 93.08% | NHS Digital [4] |
| *Distribution of diabetes by IMD quintile (IMD 1 – most deprived)* | | | |
| E† | % T1DM in IMD 1 | 20% | NHS Digital [4] |
| F† | % T2DM in IMD 1 | 24% | NHS Digital [4] |
| G = A*B*C*E | No. of patients with T1DM in IMD 1 | 45 629 | Calculated |
| H = A*B*D*F | No. of patients with T2DM in IMD1 | 729 262 | Calculated |
| I = G+H | All patients with diabetes in IMD1 | 774 890 | Calculated |
| *Distribution of DMO by IMD quintile (IMD 1 – most deprived)* | | | |
| J | Prevalence of DMO | 7.12% | Minassian, Owens [5] |
| K = I*J | N patients with DMO in IMD1 | 55 172 | Calculated |
| Abbreviations: DMO, diabetic macular oedema; IMD, index of multiple deprivation; T1DM, type 1 diabetes mellitus; T2DM, type 2 diabetes mellitus  † IMD specific input, all other inputs are the same across all IMD quintiles. IMD specific inputs are presented in Table S3. | | | |

Table S2. Eligibility criteria for faricimab and number of patients by index of multiple deprivation quintiles (IMD 1 – most deprived)

|  | **Item** | **Estimate** | **Source** |
| --- | --- | --- | --- |
| A | % with visual impairment due to DMO | 37.3% | Minassian, Owens [5] |
| A_N_ = A*K | Prevalence of visual impairment due to DMO in IMD1 | 20 593 | Calculated |
| B | % with central retinal thickness of 400 micrometres | 26.0% | NICE [6] |
| B_N_ = A_N_*B | Prevalent population with central retinal thickness of 400 micrometres in IMD1 | 5 354 | Calculated |
| C | % of prevalent population with a central retinal thickness < 400 micrometres who change to ≥ 400 micrometres each year | 8.5% | NICE [6] |
| C_N_= (A_N_-B_N_)*C | Prevalent population with a central retinal thickness < 400 micrometres who change to ≥ 400 micrometres each year in IMD1 | 1 295 | Calculated |
| D = B_N_+ C_N_ | Total eligible patients in IMD1 | 6 650 | Calculated |
| E | Estimated market share of faricimab | 15.0% | NICE [6] |
| F = D*E | Patients in IMD1 to receive faricimab | 997 | Calculated |
| Abbreviations: DMO, diabetic macular oedema; IMD, index of multiple deprivation | | | |

Table S3. Disease distributional inputs by index of multiple deprivation quintiles

|  | IMD1 (most deprived) | IMD2 | IMD3 | IMD4 | IMD5 (least deprived) | Source |
| --- | --- | --- | --- | --- | --- | --- |
| % of T1DM | 20% | 20% | 20% | 20% | 19% | NHS Digital [4] |
| % of T2DM | 24% | 22% | 21% | 18% | 15% |  |
| Patients with DMO (N=231 140) | 55 172 | 51 375 | 47 494 | 42 093 | 35 005 | Calculated |
| % of DMO | 24% | 22% | 21% | 18% | 15% | Calculated |
| Abbreviations: IMD, index of multiple deprivation; T1DM, type 1 diabetes mellitus; T2DM, type 2 diabetes mellitus | | | | | | |

**A4. Health benefits, opportunity costs and net health benefits disaggregated by index of multiple deprivation quintiles**

Figure S4. Population incremental health benefits, opportunity costs and net health benefit by IMD quintiles; (a) faricimab T&E vs ranibizumab PRN, (b) faricimab T&E vs aflibercept PRN, (c) faricimab T&E vs ranibizumab T&E, (d) faricimab T&E vs off-label bevacizumab PRN


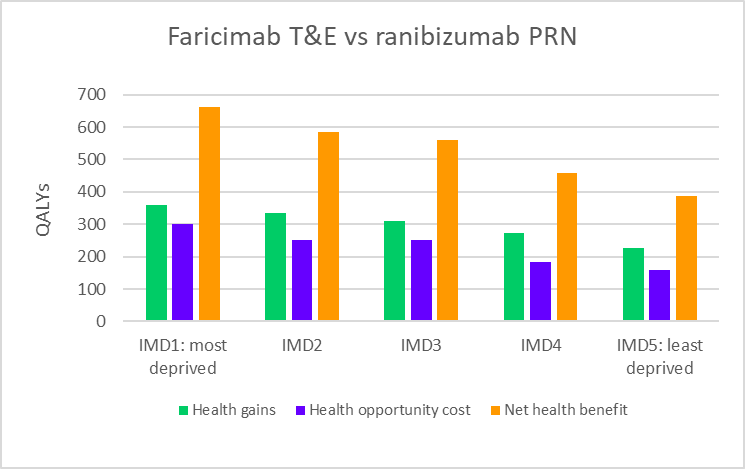


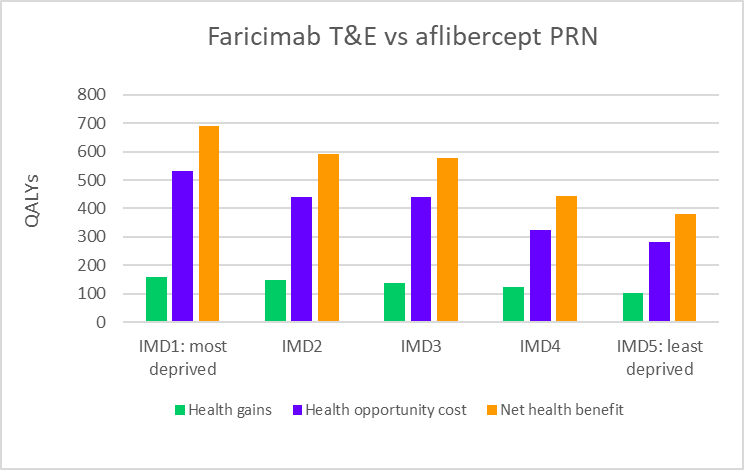


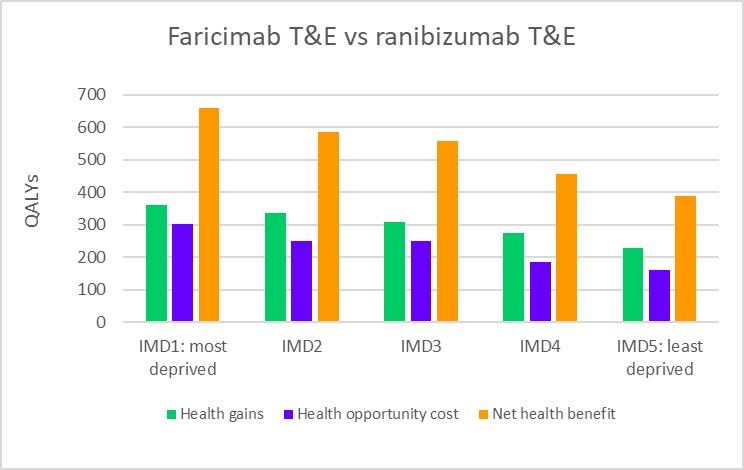


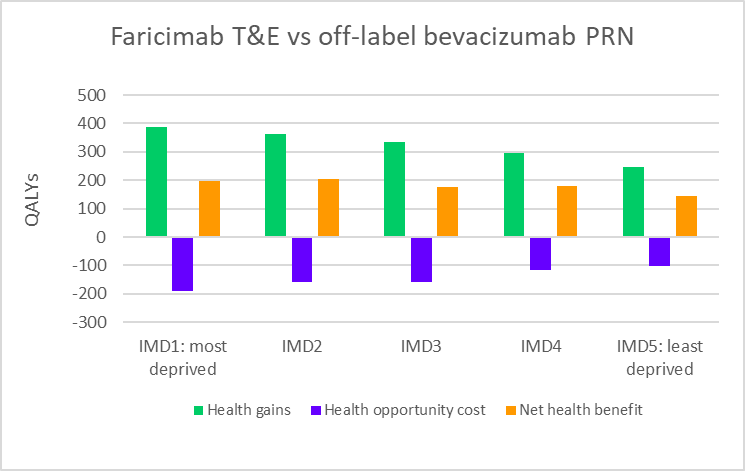


Abbreviations: IMD, index of multiple deprivation; PRN, pro re nata; QALY, quality-adjusted life-year; T&E, treat and extend

**A5. Health opportunity cost threshold scenario analysis**

At a reduced opportunity cost threshold of £15 000/QALY, the NHB, societal welfare, and equity impacts were increased when compared with ranibizumab PRN, aflibercept PRN, and ranibizumab T&E, against which faricimab T&E resulted in cost savings. Compared with off-label bevacizumab PRN, against which faricimab T&E resulted in incremental costs, the NHB, societal welfare and equity impacts were decreased (Supplementary materials, Table S4 and Figure S3). At £30 000/QALY, the results were reversed (Supplementary materials, Table S5 and Figure S4).

Table S4. Scenario analysis at an opportunity cost threshold of £15 000/QALY - Population health, societal welfare and equity impacts

|  | **Base case** | | | **Scenarios** | |
| --- | --- | --- | --- | --- | --- |
|  | **Faricimab T&E vs ranibizumab PRN** | **Faricimab T&E vs aflibercept PRN** | | **Faricimab T&E vs off-label bevacizumab PRN** | **Faricimab T&E vs ranibizumab T&E** |
| *Evaluating changes in population health (change in equity not included)* | | | | | |
| Incremental population QALE (=incremental NHB) (∆QALE*N) (1) | 3 032 QALYs | 3 359 QALYs | | 661 QALYs | 3 030 QALYs |
| *Evaluating changes in equity-weighted health (changes in health and health equity both included)* | | | | | |
| Incremental population EDEH (∆EDEH*N) (2) | 3 436 QALYs | | 3 856 QALYs | 684 QALYs | 3 434 QALYs |
| *Health equity impact* | | | | | |
| Population equity impact (incremental EDEH – incremental QALE) (2-1) | 404 QALYs | 497 QALYs | | 24 QALYs | 344 QALYs |
| Notes: QALE, quality-adjusted life-expectancy; ∆QALE, difference in QALE between post-decision and baseline; EDEH, equally distributed equivalent health; ∆EDEH, difference in EDEH between post-decision and baseline; N, England population; PRN, pro re nata; T&E, treat and extend; QALY, quality-adjusted life-year.  Scenario analysis settings: societal perspective, £15 000/QALY opportunity cost threshold, Atkinson inequality aversion parameter = 10.95 | | | | | |

Figure S5. Equity weighted population health impact with increasing inequality aversion parameter at an opportunity cost threshold of £15 000/QALY

Abbreviations: EDEH, equally distributed equivalent health; PRN, pro re nata; T&E, treat and extend

Note: Greater value of the inequality aversion parameter reflect a higher willingness to trade some of the population health to reduce health inequalities. Analysis settings: societal perspective, £15 000/QALY opportunity cost threshold

Table S5. Scenario analysis at an opportunity cost threshold of £30 000/QALY: Population health, societal welfare and equity impacts

|  | **Base case** | | | **Scenarios** | |
| --- | --- | --- | --- | --- | --- |
|  | **Faricimab T&E vs ranibizumab PRN** | **Faricimab T&E vs aflibercept PRN** | | **Faricimab T&E vs off-label bevacizumab PRN** | **Faricimab T&E vs ranibizumab T&E** |
| *Evaluating changes in population health (change in equity not included)* | | | | | |
| Incremental population QALE (=incremental NHB) (∆QALE*N) (1) | 2 268 QALYs | 2 014 QALYs | | 1 145 QALYs | 2 267 QALYs |
| *Evaluating changes in equity-weighted health (changes in health and health equity both included)* | | | | | |
| Incremental population EDEH (∆EDEH*N) (2) | 2 552 QALYs | | 2 298 QALYs | 1 245 QALYs | 2 551 QALYs |
| *Health equity impact* | | | | | |
| Population equity impact (incremental EDEH – incremental QALE) (2-1) | 284 QALYs | 285 QALYs | | 100 QALYs | 283 QALYs |
| Notes: QALE, quality-adjusted life-expectancy; ∆QALE, difference in QALE between post-decision and baseline; EDEH, equally distributed equivalent health; ∆EDEH, difference in EDEH between post-decision and baseline; N, England population; PRN, pro re nata; T&E, treat and extend; QALY, quality-adjusted life-year.  Scenario analysis settings: societal perspective, £30 000/QALY opportunity cost threshold, Atkinson inequality aversion parameter = 10.95 | | | | | |

Figure S6. Equity weighted population health impact with increasing inequality aversion parameter at an opportunity cost threshold of £30 000/QALY

Abbreviations: EDEH, equally distributed equivalent health; PRN, pro re nata; QALE, quality-adjusted life-expectancy; T&E, treat and extend

Note: Greater value of the inequality aversion parameter reflect a higher willingness to trade some of the population health to reduce health inequalities. Analysis settings: societal perspective, £30,000/QALY opportunity cost threshold

**A6. Healthcare payer perspective scenario**

Faricimab T&E is both health and equity improving (top-left quadrant) against ranibizumab PRN, aflibercept PRN and ranibizumab T&E. When compared with off-label bevacizumab PRN, faricimab T&E involved a trade-off between health maximisation and increasing health inequalities (top-right quadrant). The incremental population net health benefit remains positive although reduced compared to the base-case adopting a societal perspective. Bevacizumab, which is occasionally used in UK clinical practice despite not being licensed for the treatment of DMO, has a lower cost than faricimab and other licensed treatments. Adopting faricimab results in higher incremental costs than when compared with aflibercept or ranibizumab (Figure S7). These translate in higher health opportunity costs, which are more heavily born by the most deprived and thus a negative equity impact [7].

Figure S7. Equity-efficiency impact plane of faricimab against comparator treatment using a healthcare perspective

Abbreviations: PRN, pro re nata; QALY, quality-adjusted life-year; T&E, treat and extend

Note: analysis settings - healthcare payer perspective, £20 000/QALY opportunity cost threshold, Atkinson inequality aversion parameter = 10.95

At an opportunity-cost threshold of £20 000/QALY in a healthcare perspective (Table S6), faricimab T&E against ranibizumab T&E displayed the greatest increase in population QALE from baseline to post-decision, of 2 257 QALYs. The smallest gain with the healthcare perspective was compared with off-label bevacizumab PRN, 229 incremental QALYs from baseline. Incorporating equity-weights, the EDEH increased by 2 257 QALYs and 229 QALYs when faricimab T&E was compared against ranibizumab T&E and off-label bevacizumab PRN respectively. The greatest population equity impact of 254 QALYs was achieved compared with aflibercept PRN. Driven by the lower cost of bevacizumab, which is occasionally used off-label in clinical practice in England, a population equity impact of -38 QALYs was observed.

Table S6. The population EDEH impact at an opportunity cost threshold of £20 000/QALY with the healthcare perspective

|  | **Faricimab T&E vs ranibizumab PRN** | **Faricimab T&E vs aflibercept PRN** | **Faricimab T&E vs off-label bevacizumab PRN** | **Faricimab T&E vs ranibizumab T&E** |
| --- | --- | --- | --- | --- |
| *Evaluating changes in population health (change in equity not included)* | | | | |
| Baseline population QALE (QALE_b_*N) (1) | 3 939 130 276 QALYs | 3 939 130 276 QALYs | 3 939 130 276 QALYs | 3 939 130 276 QALYs |
| Post-decision population QALE (QALE_p_*N) (2) | 3 939 131 184 QALYs | 3 939 132,094 QALYs | 3 939 130 544 QALYs | 3 939 132 290 QALYs |
| Incremental population QALE (∆QALE*N) (3)=(2)-(1) | 908 QALYs | 1 817QALYs | 268 QALYs | 2 014 QALYs |
| *Evaluating changes in equity-weighted health (changes in health and health equity both included)* | | | | |
| Baseline population EDEH (equity weighted QALE) (EDEH_b_*N) (4) | 3 860 269 446 QALYs | 3 860 269 446 QALYs | 3 860 269 446 QALYs | 3 860 269 446 QALYs |
| Post-decision population EDEH (EDEH_p_*N) (5) | 3 860 270 423 QALYs | 3 860 271 518 QALYs | 3 860 269 676 QALYs | 3 860 271 704 QALYs |
| Incremental population EDEH (∆EDEH*N) (6) | 977 QALYs | 2 071 QALYs | 229 QALYs | 2 257 QALYs |
| *Health equity impact* | | | | |
| Population equity impact (incremental EDEH – incremental QALE) (6-3) | 69 QALYs | 254 QALYs | -38 QALYs | 243 QALYs |
| Abbreviations : QALE, quality-adjusted life-expectancy; ∆QALE, difference in QALE between post-decision and baseline; EDEH, equally distributed equivalent health; ∆EDEH, difference in EDEH between post-decision and baseline; N, England population; PRN, pro re nata; T&E, treat and extend; QALY, quality-adjusted life-year.  Notes : Scenario analysis settings - healthcare payer perspective, £20 000/QALY opportunity cost threshold, Atkinson inequality aversion parameter = 10.95 | | | | |

**A7. References**

1. Bührer, C., Paling, T., Gale, R. et al. Cost-Effectiveness of Faricimab in the Treatment of Diabetic Macular Oedema (DMO): A UK Analysis. PharmacoEconomics Open (2024). https://doi.org/10.1007/s41669-023-00465-4.

2. ONS, *Sex by single year of age 2021*. 2022: Office for National Statistics.

3. NHS Digital. *Data on file: Quality and Outcomes Framework, 2021-22 (September 2022)*. 2022 [cited 2023 14 April 2023]; Available from: <https://digital.nhs.uk/data-and-information/publications/statistical/quality-and-outcomes-framework-achievement-prevalence-and-exceptions-data/2021-22>.

4. NHS Digital. *National Diabetes Audit*. 2022; Available from: <https://digital.nhs.uk/data-and-information/clinical-audits-and-registries/national-diabetes-audit>.

5. Minassian, D.C., D.R. Owens, and A. Reidy, *Prevalence of diabetic macular oedema and related health and social care resource use in England.* Br J Ophthalmol, 2012. **96**(3): p. 345-9.

6. NICE. *TA820 Resource impact template: Brolucizumab for treating diabetic macular oedema*. 2022; Available from: <https://www.nice.org.uk/guidance/ta820/resources>.

7. Love-Koh, J., et al., *Estimating social variation in the health effects of changes in health care expenditure.* Med Decis Making, 2020. **40**(2): p. 170-182.
